# Variability of Diagnostic Outcomes in Nasal Allergen Challenge: The Role of Combined Subjective and Objective Indicators

**DOI:** 10.1002/clt2.70124

**Published:** 2025-11-19

**Authors:** Yu Zhang, Yu Song, Xu Zhang, Jingyun Li, Zengxiao Zhang, Lin Xi, Luo Zhang, Yuan Zhang

**Affiliations:** ^1^ Department of Allergy Beijing Tongren Hospital Capital Medical University Beijing China; ^2^ Department of Otolaryngology Head and Neck Surgery Beijing Tongren Hospital Capital Medical University Beijing China; ^3^ Beijing Laboratory of Allergic Diseases and Beijing Key Laboratory of Nasal Diseases Beijing Institute of Otolaryngology Beijing China; ^4^ Laboratory for Clinical Medicine Capital Medical University Beijing China

**Keywords:** allergic rhinitis, *Dermatophagoides farinae*, diagnostic tools, nasal allergen challenges, serum‐specific IgE

## Abstract

**Background:**

Nasal allergen challenge (NAC) is currently the only available test to confirm nasal responsiveness to allergens and is a core diagnostic tool for allergic rhinitis. NAC is currently diagnosed by combining existing subjective measures and objective assessment methods in pairs to assess outcomes, based on a strong positive result for a single item or a moderately positive result for a combination of two items. However, there are few data on the diagnostic outcomes and characteristics of its various diagnostic combinations.

**Objective:**

To evaluate the differences and characteristics of various existing diagnostic methods (combinations of subjective and objective indicators) for NAC.

**Method:**

During January 2023–December 2024, patients with history of chronic rhinitis (CR) who experienced nasal symptoms upon exposure to dust, as well as healthy controls, were enrolled. Demographic and clinical data were collected, followed by a NAC with *Dermatophagoides farinae* (Df). Total nasal symptom score (TNSS), visual analog scales (VAS), acoustic rhinometry (AcRh), Active anterior rhinomanometry (AAR) and 4‐phase‐rhinomanometry (4PR) were used for assessment before and after NAC. The diagnostic results, minimum NAC concentration at positive trigger, and type of positive response (subjective or objective) were recorded.

**Result:**

A total of 180 patients and 133 healthy controls were enrolled in the study. Significant differences in positive rates were observed across the six diagnostic evaluation combinations (*p* = 0.001). The combination of subjective TNSS evaluation with objective AAR assessment demonstrated the highest positive rate among patients (55.6%), whereas the combination of subjective VAS evaluation with objective AcRh assessment yielded the lowest positive rate (31.7%). Furthermore, when diagnostic combinations triggered positive results, they favored strong objective positivity.

**Conclusion:**

The diagnostic results derived from pairwise combinations of various subjective measurements and objective assessments exhibit variability, with objective assessments demonstrating a higher propensity to yield positive results compared to subjective measurements.

## Graphical abstract

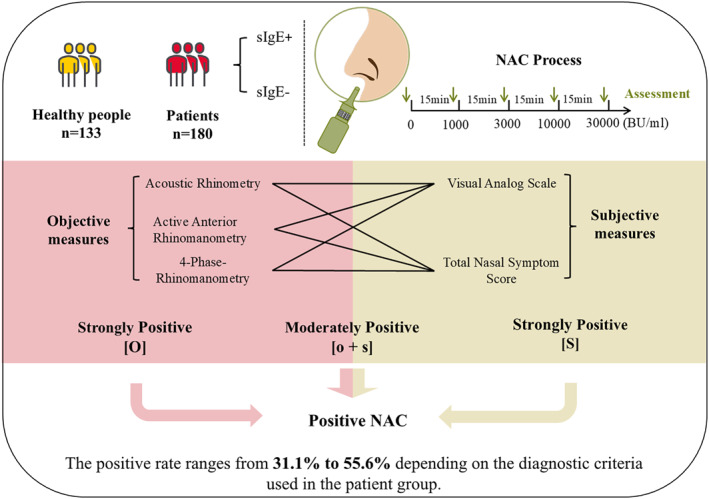

## 1 Introduction

Allergic Rhinitis (AR) is an IgE‐mediated inflammatory disease of the nasal mucosa triggered by exposure to environmental allergens. It is among the most prevalent chronic diseases globally [1]. For many years, the diagnosis of AR has primarily relied on a clinical history of allergic symptoms in combination with in vivo and in vitro IgE‐specific tests, such as the Skin Prick Test (SPT) and serum specific IgE (sIgE) testing. These diagnostic tools have become the cornerstone of clinical practice. However, a notable diagnostic gap persists: a substantial number of patients exhibiting typical AR symptoms test negative on these assessments and have historically been classified as having Non‐Allergic Rhinitis (NAR). In recent years, with advances in research, a distinct nasal inflammatory phenotype—Local Allergic Rhinitis (LAR)—has gained recognition [2, 3]. LAR is characterized by an allergic reaction localized to the nasal mucosa, with patients exhibiting Th2‐driven inflammatory responses and classic nasal symptoms, despite the absence of detectable peripheral sIgE and negative SPT results. The underlying mechanism is believed to involve local sIgE production and the presence of sensitized immune cells within the nasal mucosa [4, 5].

The nasal allergen challenge (NAC) refers to the controlled administration of allergens onto the nasal mucosa under standardized conditions, aiming to assess whether it can elicit the principal symptoms of AR. By simulating the upper respiratory tract's reaction to natural allergen exposure within a regulated setting, NAC is currently the only available test to confirm nasal reactivity to allergens [6, 7]. It is regarded as the “gold standard” for diagnosing AR, especially LAR, and also has considerable value in evaluating treatment responses and other aspects [4, 8, 9].

The current approach to NAC diagnosis is to recommend the use of existing subjective and objective assessment methods. Subjective evaluations included visual analog scales (VAS), Lebel score, Linder score, and total nasal symptom score (TNSS), whereas objective assessments encompassed peak nasal inspiratory flow (PNIF), acoustic rhinometry (AcRh), active anterior rhinomanometry (AAR), and four‐phase nasal resistance (4PR). The outcome of the NAC is determined by a combined evaluation of subjective and objective parameters. A positive NAC result is concluded if one parameter shows strong positivity or if both demonstrate moderate positivity, indicating that the patient has developed a nasal allergic reaction following allergen exposure [10].

However, currently, different medical institutions employ varying combinations of subjective assessments and objective indicators. Relying solely on a single indicator—such as a purely subjective score—may introduce patient‐related bias, while an exclusive reliance on objective indicators may inadequately reflect the patient's current symptomatology. Furthermore, despite being the most authoritative diagnostic criterion for NAC, it represents a consensus derived from expert opinion rather than being substantiated by sufficient empirical research evidence [11].

This study, for the first time, systematically compared various combinations of subjective and objective diagnostic criteria, rather than relying on a single indicator or combination. The aim was to evaluate the diagnostic efficacy of NAC from multiple perspectives, analyze whether different combinations produce distinct diagnostic outcomes, and identify their specific characteristics in terms of diagnostic performance, with the goal of providing evidence‐based support for optimizing the diagnostic criteria for NAC.

## 2 Methods

### 2.1 Study Population

Participants were recruited from January 2023 to December 2024. The study included two groups: the patient group, consisting of individuals who experienced perennial nasal symptoms upon exposure to dust and were either diagnosed with or suspected of having an allergy to dust mites, and the healthy control group, comprising individuals without any symptoms of rhinitis. All participants provided written informed consent. The study protocol was approved by the Ethics Committee of Beijing Tongren Hospital, Capital Medical University.

All participants in the patient groups were required to meet the following inclusion criteria: (1) Perennial symptoms related to chronic rhinitis, such as nasal obstruction, rhinorrhea, sneezing, nasal itching, etc. (2) Dust mites are identified or suspected allergens‐specific SPT positive or sIgE≥ 0.35kU/L, or SPT and sIgE negative but with a history of perennial chronic rhinitis and complaint of nasal symptoms whenever exposed to dust; (3) The patient should be in a period of symptom remission or be asymptomatic (Table S1); (4)The following drugs have not been used or have been discontinued for a sufficient period of time: topical antihistamines (4–5 days), topical corticosteroids (2–4 days), topical mast‐cell stabilizers (7–21 days), systemic antihistamines (7 days), systemic corticosteroids (14–21 days), systemic NSAIDs (7 days), leukotriene modifiers (no specific recommendation), topical or systemic decongestants (2 days), tricyclic antidepressants (14–21 days), and clonidine or other centrally acting agents (21 days); (5) No history of nasal surgery or 6–8 weeks after nasal surgery; (6) Do not smoke or consume stimulating beverages (such as alcohol, coffee, milk tea, etc.) 24–48 h before the examination.

Participants with any of the following criteria were excluded from the patient group: (1) uncontrolled asthma; (2) age ≤ 14 years; (3) presence of nasal conditions that could interfere with the study, such as chronic rhinosinusitis, nasal polyps, severe nasal obstruction, or perforated nasal septum; (4) women who are pregnant, lactating, or in the preconception phase; (5) a history of severe allergic reactions or anaphylactic shock; (6) severe chronic obstructive pulmonary disease (COPD) or other severe cardiopulmonary diseases contraindicating the use of epinephrine; (7) active or onset phase of other serious systemic diseases, including malignant tumors or autoimmune disorders.

The healthy control group should meet the following conditions: no nasal symptoms or signs, no history of allergic diseases, negative serum sIgE of all allergens < 0.35 kU/L and total immunoglobulin E (tIgE) < 60 kU/L.

### 2.2 Serum tIgE and sIgE Measurement

Serum tIgE and sIgE levels were measured using immunofluorescence with a fully automatic immunofluorescence analyzer (Thermo Phadia 1000). A threshold value of 0.35 kU/L was used as the cutoff for determining positive sIgE results.

### 2.3 Nasal Allergen Challenge

All participants are required to acclimatize for 20–30 min in the room where the NAC will be performed before initiating the challenge.

All participants underwent NAC with *Dermatophagoides farinae* (Df): Participants who met the inclusion criteria recorded baseline characteristics, including age, gender, disease duration, family history of atopic diseases, and concomitant diseases, etc.

Subjective and objective assessments were conducted at baseline, 15 min after the administration of normal saline, and 15 min following the administration of each concentration of Df allergen extract (60,000 BU/mL, ALK‐Abelló).

The subjective evaluation indicators were TNSS and VAS to quantify the clinical symptoms. TNSS is a 12‐point scale commonly used to evaluate nasal symptoms. It is calculated as the sum of scores for four individual symptoms: rhinorrhea, nasal obstruction, sneezing, and nasal itching. Each symptom is rated on a scale from 0 to 3 (0 = none, 1 = mild, 2 = moderate, or 3 = severe) [12]. According to the ARIA guidelines, VAS consists of a vertical line scaled from 0 to 100 mm, allowing patients to subjectively report the severity of nasal symptoms. This method provides a clear and straightforward means of evaluating the intensity of clinical symptoms [13]. Objective evaluation indicators were AcRh, AAR and 4PR. The total nasal volume (TNV) and minimum cross‐sectional area (MCA) under AcRh, total nasal resistance (TNR) under 4PR, and total nasal flow at 150 Pa under AAR were recorded [5, 10, 14].

To exclude nonspecific nasal hyperreactivity (NHR), bilateral nasal tests were conducted using normal saline prior to NAC. If the reaction under the control agent is ≥ 50% of a positive reaction, we recommend that the test is halted. If the control solution causes < 50% of the positivity criteria, NAC could be continued. Subsequently, the allergen concentration was progressively increased during the NAC procedure. Initially, NAC was conducted using Df extract solutions at a concentration of 1000 BU/mL, with subsequent incremental increases to 3000 BU/mL, 10,000 BU/mL, and finally 30,000 BU/mL [15]. If none of the concentrations met the criteria for a positive response, the evaluation was repeated after a one‐hour interval to monitor for any late‐phase reaction [16].

Positive criteria for NAC: the 2018 NAC position paper from the European Academy of Allergy and Clinical Immunology (EAACI) recommended employing both existing subjective and objective assessment methods. Strong positive in a single item or moderately positive in both subjective and objective combinations [10, 12], as detailed in Table 1.

**TABLE 1:** Diagnostic criteria for NAC.

Method	Clearly positive (S; O)	Moderately positive (s; o)
Subjective measures		
TNSS	Increase of ≥ 5 points	Increase of ≥ 3 points
VAS	Symptoms ≥ 55 mm	Symptoms ≥ 23 mm
Objective measures		
AAR	Flow decrease of ≥ 40% at 150 Pa	Flow decrease of ≥ 20% at 150 Pa
AcRh	CSA‐2 decrease of ≥ 40%	Decrease in sum of 2–6 cm^3^ ≥ 27% bilaterally
4PR	≥ 40% increase in logarithmic (lg) effective resistance	≥ 20% increase in logarithmic (lg) effective resistance

Abbreviations: 4PR: 4‐phase‐rhinomanometry; AAR: Active Anterior rhinomanometry; AcRh: Acoustic rhinometry; o + s: moderate increase in two criteria (objective and subjective measurement); O: Strong increase in objective measurement; S: Strong increase in subjective symptoms; TNSS: total nasal symptom score; VAS: Visual analog scale.

### 2.4 Statistical Analysis

SPSS 26 (SPSS IBM Corp., Armonk, NY, USA) was employed for statistical analysis. Quantitative data were first subjected to normality tests. For variables with normal distribution, results were expressed as mean ± standard deviation ($\overset{-}{x}$± s), and intergroup comparisons were performed using Student's t‐test. Non‐normally distributed variables were summarized using median and interquartile range (IQR), and group differences were assessed using non‐parametric tests (Kruskal‐Wallis test or Mann‐Whitney *U* test). Qualitative data were described in terms of frequency and proportion, and intergroup comparisons were conducted using the chi‐square (X^2^) test. For each diagnostic combination, we calculated the discriminative indicators (sensitivity (SE), specificity (SP) and Youden's Index), predictive indicators (positive and negative predictive values (PPV and NPV, respectively)), as well as the consistency rate between serological results and NAC, which was assessed using the Kappa index. All statistical tests were two‐sided, and a *p*‐value < 0.05 was considered statistically significant.

## 3 Results

### 3.1 Clinical and Analytical Features of Study Individuals

A total of 313 participants were enrolled in this study, including 180 patients diagnosed with chronic rhinitis and 133 healthy controls. The median age of the patient group was 34 years (IQR: 30–40.75), with 80 males (44.4%) and 100 females (55.6%). The healthy control group had a median age of 47 years (IQR: 31–56), consisting of 43 males (32.87%) and 90 females (67.7%). Serum tIgE levels in patients were significantly higher than those in the healthy control group (*p* < 0.001).

Based on serum Df‐sIgE, the patient population was further categorized into an sIgE‐positive group and an sIgE‐negative group. There were 49 cases in the sIgE‐positive group and 133 cases in the sIgE‐negative group. Baseline characteristics are presented in detail in Table 2. A significant statistical difference was observed between the two groups in terms of tIgE levels, with the seropositive group showing significantly higher values (*p* < 0.001). Additionally, the disease duration was significantly longer in the sIgE‐positive group (*p* = 0.037). Moreover, compared to patients in sIgE‐positive group, those in sIgE‐negative group exhibited more persistent symptoms (*p* = 0.011). No statistically significant differences were found between the groups regarding nasal or ocular symptom scores.

**TABLE 2:** Baseline characteristics in sIgE‐positive and sIgE‐negative patients.

Characteristic	sIgE≥ 0.35 (*n* = 49)	sIgE< 0.35 (*n* = 131)	*p*
Age, mean (years), median (IQR)	34 (29–41)	34 (30–39)	0.500
Gender			
Female	22 (44.9%)	78 (59.5%)	0.078
Male	27 (55.1%)	53 (40.5%)	
Total IgE (Ku/L), median (IQR)	308.50 (88.50–684.50)	73.75 (29.68–116.00)	< 0.001
Family atopy	15 (30.6%)	38 (29%)	0.833
Smoking or drinking history	12 (24.5%)	35 (26.7%)	0.850
Disease duration (years), median (IQR)	84 (42–194)	60 (30–120)	0.037
Comorbidity			
Asthma	2 (4.1%)	19 (14.5%)	0.053
Allergic dermatitis	12 (24.5%)	26 (19.8%)	0.497
Allergic conjunctivitis	13 (26.5%)	24 (18.3%)	0.225
Food allergy	6 (12.2%)	6 (4.6%)	0.067
Drug allergy	2 (4.1%)	17 (13.0%)	0.084
Symptom characteristics			
Persistent	22 (44.9%)	86 (65.6%)	0.011
Intermittent	27 (55.1%)	45 (34.4%)	
Nasal symptoms			
Nasal itching	19 (38.8%)	50 (38.2%)	0.941
Sneezing	9 (18.4%)	22 (16.8%)	0.803
Nasal obstruction	26 (53.1%)	76 (58.0%)	0.551
Rhinorrhea	24 (49.0%)	62 (47.3%)	0.843
Eye symptoms			
Eye itching	14 (28.6%)	40 (30.5%)	0.789
Tearing	7 (14.3%)	19 (14.5%)	0.970

Abbreviation: IgE, immunoglobulin E.

### 3.2 Distribution of the NAC Results for Different Diagnostic Combinations

**FIGURE 1:**
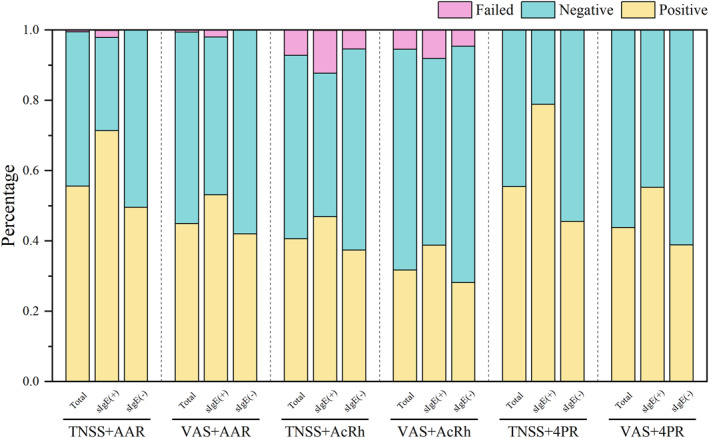
Distribution of results among patients according to various diagnostic combinations of NAC. 4PR: 4‐phase‐rhinomanometry; AAR: Active Anterior rhinomanometry; AcRh: Acoustic rhinometry; sIgE, specific immunoglobulin E; TNSS: total nasal symptom score; VAS: Visual analog scale.

Among the 180 patients included in the study, statistically significant differences were observed in the positive rates across various diagnostic combinations (*p* < 0.001). The highest positive rate was achieved when the TNSS subjective assessment was combined with the AAR objective assessment (55.6%), while the lowest positive rate was recorded when the VAS subjective assessment was paired with the AcRh objective assessment (31.7%) (Figure 1).

We divided the patients into serum sIgE‐positive and sIgE‐negative groups based on serum Df sIgE levels. We further analyzed differences across diagnostic criteria and found that, in the sIgE‐positive group, the highest positive rate was achieved by combining subjective evaluation using TNSS with objective assessment via 4PR (78.9%), followed by the combination of TNSS and AAR (71.4%). The lowest positive rate was observed when using VAS combined with AcRh (38.8%). In the sIgE‐negative group, the combination of TNSS and AAR yielded the highest positive rate (49.6%), while the combination of VAS and AcRh showed the lowest positive rate (28.2%).

Meanwhile, we also performed the same outcome analysis in the healthy control group. In the objective evaluation of AcRh, whether assessed in combination with TNSS or VAS scores, 8 healthy individuals (6.0%) exhibited a positive response to NAC, and 4 individuals (3.0%) demonstrated sensitivity to normal saline, indicating a hyperreactive state of the nasal mucosa. Similarly, in the 4PR objective assessment, one healthy participant also showed a positive reaction to NAC.

**FIGURE 2:**
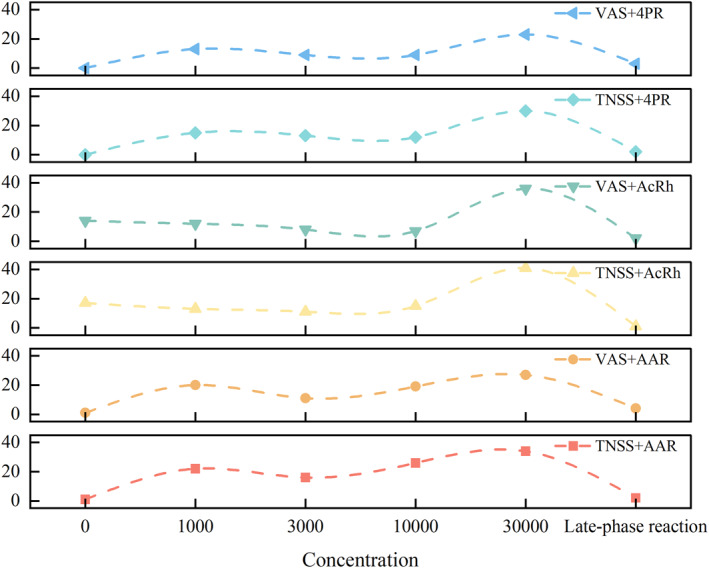
Performance of various diagnostic combinations of NAC in terms of concentration. 4PR: 4‐phase‐rhinomanometry; AAR: Active Anterior rhinomanometry; AcRh: Acoustic rhinometry; TNSS: total nasal symptom score; VAS: Visual analog scale.

Furthermore, with regard to the concentration of the excitation solution that triggers positive responses, no statistically significant difference was observed in the performance of positive samples across different combinations (*p* = 0.265), and the highest proportion of positive responses was recorded when the excitation solution concentration was 30,000 BU/mL (Figure 2). However, upon analyzing the two patient groups classified by serological status, we found that certain patients in the sIgE‐negative group exhibited a delayed response, which was absent in the sIgE‐positive group.

### 3.3 Analysis of Subjective Score and Objective Evaluation of NAC Diagnosis

**FIGURE 3:**
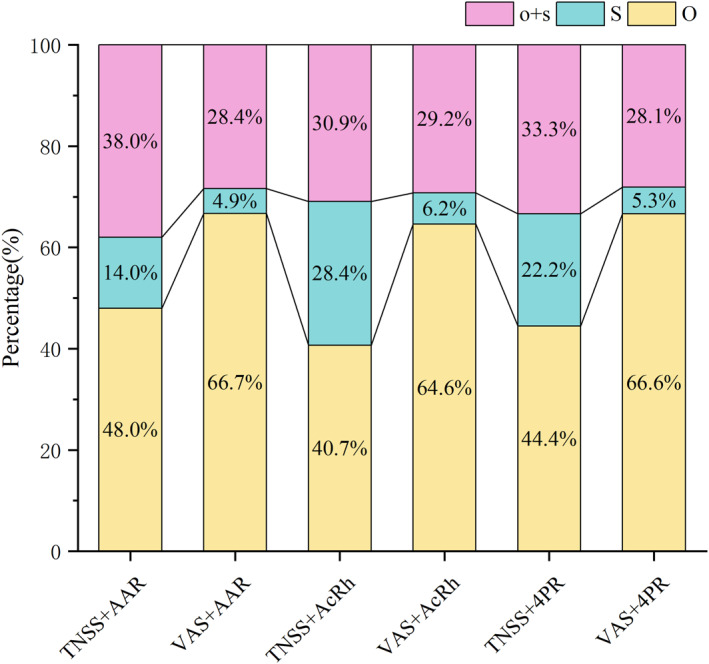
Distribution of diagnostic criteria for positive samples. 4PR: 4‐phase‐rhinomanometry; AAR: Active Anterior rhinomanometry; AcRh: Acoustic rhinometry; o + s: moderate increase in two criteria (objective and subjective measurement); O: Strong increase in objective measurement; S: Strong increase in subjective symptoms; TNSS: total nasal symptom score; VAS: Visual analog scale.

We observed that all false positive phenomena occurring in the healthy population were attributable to objective strong positive diagnoses. This indicates that our simple objective evaluation method may be overly sensitive, potentially leading to an increased rate of false positives. To further investigate this issue, we analyzed the distribution of diagnostic criteria for positive samples across different diagnostic combinations (Figure 3). Specifically, we examined the proportions of simple objective strong positives, simple subjective strong positives, and combined subjective and objective positives within each diagnostic combination. It was found that although there were significant statistically significant differences in the proportion of diagnostic criteria of different subjective and objective diagnostic combinations (*p* < 0.001), each diagnostic combination tended to be mainly objective strong positivity, followed by the combination of subjective and objective, which indicated that the positive proportion of NAC triggered by a strongly positive subjective symptom score was the lowest. This also applies to the analysis of the respective outcomes of the two patient groups categorized based on serum sIgE levels.

### 3.4 Evaluation of the Diagnostic Efficiency of the NAC

We selected patients with serum Df sIgE levels ≥ 0.35 kU/L from the patient population and compared them with the recruited healthy controls. Using serological diagnosis and clinical symptoms as the reference standards, we analyzed the diagnostic performance of each combination of diagnostic criteria (Table 3). Among them, the combined TNSS + 4PR and TNSS + AAR showed a high level of agreement with the serological diagnostic results (Kappa = 0.837, *p* < 0.001; Kappa = 0.798, *p* < 0.001). Meanwhile, compared with other combinations, these two combinations exhibited higher sensitivity and specificity. However, the VAS + AcRh combination performed the worst in both consistency with serological diagnosis and diagnostic efficacy.

**TABLE 3:** Diagnostic accuracy of NAC (Allergy subject vs. Health).

Diagnostic combination	Kappa index	*p*‐value	SE	SP	Youden's index	PPV	NPV
TNSS + AAR	0.798	< 0.001	72.9%	100%	0.73	100%	91.1%
VAS + AAR	0.635	< 0.001	54.2%	100%	0.54	100%	85.8%
TNSS + AcRh	0.521	< 0.001	53.5%	93.8%	0.47	74.2%	85.8%
VAS + AcRh	0.414	< 0.001	42.2%	93.8%	0.36	70.4%	82.3%
TNSS + 4PR	0.837	< 0.001	78.9%	99.2%	0.78	96.8%	94.2%
VAS + 4PR	0.641	< 0.001	55.3%	99.2%	0.55	95.5%	88.4%

Abbreviations: 4PR: 4‐phase‐rhinomanometry; AAR: Active Anterior rhinomanometry; AcRh: Acoustic rhinometry; NPV: negative predictive value; PPV: positive predictive value; SE: sensitivity; SP: specificity; TNSS: total nasal symptom score; VAS: Visual analog scale.

## 4 Discussion

NAC is a valuable tool for monitoring the response of the human nasal mucosa to allergen exposure and can effectively guide the treatment of rhinitis. Even in today's era of widespread application of molecular allergen diagnosis, NAC still holds an irreplaceable position. By simulating the natural exposure process on the target organ, it has become the “gold standard” for confirming the causal relationship between allergens and clinical symptoms and is currently the only method available for diagnosing LAR. Moreover, its application is supported by evidence demonstrating high safety and reproducibility [17, 18]. Studies have shown that NAC also has potential value in the prevention and monitoring of the occurrence and development of lower respiratory tract diseases. Additionally, NAC has been widely used in pathogenesis research, contributing to a deeper understanding of various aspects of the pathophysiology of AR. These include the acute release of mast cell mediators during allergen exposure, local infiltration of inflammatory cells, type 2 inflammatory responses, the production of related cytokines and chemokines, and the development of nasal hyperresponsiveness [19, 20]. In summary, NAC is a powerful complementary tool to molecular diagnosis.

This study presents the first head‐to‐head comparison of multiple diagnostic combinations within the same population undergoing NAC, systematically evaluating pairwise combinations of two subjective scores (TNSS, VAS) and three objective indicators (4PR, AAR, AcRh). Our results demonstrate considerable variability in positivity rates depending on the diagnostic paring applied, underscoring the choice of combination significantly influences the outcomes of NAC.

Consistent with previous reports, we confirm that subjective endpoints alone are insufficiently unreliable due to individual variability and patient bias. Additionally, among the healthy control group, regardless of whether AcRh was combined with the TNSS or the VAS score, false positive results were observed in 8 individuals (6.0%), representing the highest proportion across all groups. Moreover, all these false positive cases were classified as objectively strongly positive. This phenomenon suggests that objective diagnostic criteria are more likely to result in a positive determination than subjective scoring methods, which may result in a higher rate of false positives.

Importantly, this study goes beyond the traditional “single gold standard” research paradigm. Previous studies have predominantly focused on a single diagnostic combination, such as the combination of VAS and PNIF utilized in the study by Kim et al. [21], and this study confirmed through analysis that various diagnostic combinations each possess distinct clinical value. For instance, when serumology and clinical symptoms are used as the gold standard, the TNSS + 4PR and TNSS + AAR combinations achieve the best balance between sensitivity and specificity, with the TNSS + 4PR combination having the highest Youden index (0.78) and a positive rate of 78.9%; while the combination of VAS and AAR had high specificity but relatively low sensitivity. These findings reinforce the multi‐dimensional nature of NAC diagnosis and provide evidence‐based guidance for combined selection in clinical practice and future guideline updates.

This study observed that the average age of the healthy control group was significantly higher than that of the patient group, and the proportion of females was also significantly greater (*p* < 0.05). These differences may be attributed to variations in population recruitment sources and intentions. However, since the primary objective of this study was to evaluate differences in diagnostic outcomes and efficacy across various combinations of NAC, rather than to directly compare intergroup differences, and given that all healthy controls met strict inclusion and exclusion criteria, these demographic discrepancies do not compromise the independent assessment of diagnostic performance.

Furthermore, this study has certain limitations. Specifically, the objective and subjective assessments recommended by EAACI each include four types. However, due to limitations in research equipment and subject recruitment, this study employed only the most commonly used subjective evaluation tools in clinical practice—namely, TNSS and VAS—and did not include the objective indicator PNIF. Nevertheless, these limitations did not have a substantial impact on the overall study design. In terms of sample size, although the current study generally meets basic statistical requirements, the statistical power remains insufficient for certain subgroup analyses—particularly within the serum sIgE‐positive group. This limitation affects the stability and accuracy of the results to some extent, especially in the evaluation of diagnostic performance. Moreover, cases that yielded failed results in NAC were excluded from the analysis, potentially leading to an underestimation of the response characteristics in highly sensitive individuals. To improve the reliability of future findings, it is advisable for subsequent studies to aim at increasing the overall sample size.

Notably, the EAACI position paper published in 2018 clearly stated that the use of freeze‐dried, glycerin‐free standardized preparations dedicated to the nasal cavity should be preferred to reduce nonspecific irritation due to preservatives or glycerin [12]. However, the American Academy of Allergy and Immunology task Force report also stated that investigational NAC using glycerylated skin testing or immunotherapy formulations has become a common and acceptable practice in the absence of dedicated nasal challenge reagents [12]. Commercial glycerin‐free NAC solution was not available in the country where this study was conducted. Therefore, a commercial house dust mite SPT solution (60,000 BU/mL, ALK‐Abelló) was selected as the challenge reagent based on comprehensive consideration of reagent availability, potency traceability and methodological feasibility. Similarly, for example, Joo et al. [22] used the standardized dust mite extract (containing glycerol) diluted in proportion to perform NAC when they established the “Korean standardized nasal challenge process”, and verified its safety and repeatability. We fully recognize that the use of glycerin‐containing preparations in nasal provocation tests remains somewhat controversial. Glycerol, as an antiseptic and stabilizer, can improve extract stability, but its hyperosmolarity and viscosity may lead to transient physical irritation. Based on this, the possible stimulation response caused by glycerol has been discussed as a methodological limitation in this study, and future studies are recommended to use lyophilized or glycerin‐free standardized preparations when conditions permit, or to advance the production of standardized products of NAC challenge solutions to further control for potential confounders. To sum up, this study demonstrates that different combinations yield significantly varying diagnostic outcomes. Notably, although diagnostic strategies dominated by objective strong positives underscore the high sensitivity of the detection technology, their clinical applicability should be comprehensively assessed after carefully considering the potential risk of false positives. This necessitates the establishment of an evaluation system that differentiates “technically true positive” from “clinically true positive,” and promotes a shift in diagnostic criteria from the traditional “single cut‐off value” model to precision diagnosis based on individual patient characteristics. This transformation is expected to enhance the accuracy of NAC diagnosis, thereby providing a more reliable evidence‐based foundation for optimizing diagnostic and therapeutic strategies and advancing mechanistic research.

## Author Contributions

Concept and design: Luo Zhang and Yuan Zhang. Acquisition, analysis, or interpretation of data: Yu Zhang, Yu Song, Xu Zhang, Jingyun Li, and Lin Xi. Drafting of the manuscript: Yu Zhang, Yu Song, Luo Zhang, and Yuan Zhang. Critical review of the manuscript for important intellectual content: Luo Zhang, and Yuan Zhang. Statistical analysis: Yu Zhang, Yu Song, Xu Zhang, Zengxiao Zhang and Jingyun Li. Obtained funding: Luo Zhang and Yuan Zhang. Administrative, technical, or material support: Luo Zhang and Yuan Zhang. Supervision: Luo Zhang and Yuan Zhang.

## Funding

This work was supported by grants from National Key R&D Program of China (Grant 2022YFC2504100), the Program for the Changjiang Scholars and Innovative Research Team (Grant IRT13082), Natural Science Foundation of China (Grants 82471132 and 82071022) and Beijing Hospitals Authority Clinical Medicine Development of Special Funding (Grant ZLRK202303).

## Ethics Statement

This study was approved by the ethics committee of Beijing Tongren Hospital of Capital Medical University (TREC2023‐KY046). All patients provided informed consent, and the study protocol complied with the ethical guidelines of the Declaration of Helsinki.

## Conflicts of Interest

The authors declare no conflicts of interest.

## Supporting information


**Table S1**: The baseline values of all measurement indicators for all subjects.

## Data Availability

The data that support the findings of this study are available on request from the corresponding author. The data are not publicly available due to privacy or ethical restrictions.
