# Supplementary material for: Variability of Diagnostic Outcomes in Nasal Allergen Challenge: The Role of Combined Subjective and Objective Indicators
Source: Clin Transl Allergy. 2025 Nov 19;15(11):e70124. doi: 10.1002/clt2.70124 (PMC12628074; doi:10.1002/clt2.70124)
Supplement: Supplementary file 1 — Table S1: The baseline values of all measurement indicators for all subjects. [file CLT2-15-e70124-s001.docx]

Supplementary Table S1. The baseline values of all measurement indicators for all subjects

| Measurement indicators | Baseline Value/ median (IQR) |
| --- | --- |
| TNSS | 0.0 (0.0 - 2.0) |
| VAS | 0.00 (0.00 - 10.25) |
| Minimum Cross-Sectional Area (cm²) | 0.90 (0.69 - 1.17) |
| Total Nasal Volume (cm³) | 8.96 (7.55 - 11.21) |
| Total Nasal Flow (ml/s) | 677.20 (508.91 - 898.20) |
| Total Nasal Resistance (Pa) | 0.34 (0.28 - 0.42) |

TNSS: total nasal symptom score; VAS: Visual analog scale
